# From Michotte Until Today: Why the Dichotomous Classification of Modal and Amodal Completions Is Inadequate

**DOI:** 10.1177/2041669519841639

**Published:** 2019-05-17

**Authors:** Tom R. Scherzer, Franz Faul

**Affiliations:** Institute of Psychology, Kiel University, Germany

**Keywords:** amodal completion, classification, modal completion, perception, taxonomy

## Abstract

The distinction between modal and amodal completion is ubiquitous in the perception literature. It goes back to the seminal publication “Les compléments amodaux des structures perceptives” by A. Michotte, G. Thinès, and G. Crabbé (Publications Universitaires de Louvain: Louvain) in 1964. We review and discuss this work in this article and show commonalities and differences to today’s view. We then argue that the dichotomous distinction between modal and amodal completions is problematic in phenomenological, empirical, logical, and theoretical terms. Finally, we propose alternative criteria allowing for a more differentiated classification scheme for completion phenomena. This scheme seems to be consistent with all known empirical findings and can also be generalized to nonvisual domains of perception.

## Introduction

An important prerequisite for systematic, focused research in all scientific disciplines is the organization of research objects in a hierarchical system of classes whose elements share essential characteristics. In perception psychology, there are modal and amodal completion phenomena, which belong to the class of perceptual completion phenomena. However, this common classification seems problematic to us for various reasons, as we will discuss in this article. Pessoa, Thompson, and Noë (1998) presented a much-noticed taxonomy of completion phenomena, and there are some more suggestions in the literature (e.g., Dresp, 1998; Weil & Rees, 2011), but our criticism addresses the underlying dichotomous distinction between “modal” and “amodal” and is thus more fundamental.

Phenomena of perceptual completion demonstrate the constructive character of perception. In this context, the term “perceptual completion” typically refers to mental processes that lead to the perception of an entire object despite only partial stimulation, i.e., even though parts of the perceptually represented object have no actual correspondence, or “local counterparts” (van Lier & Gerbino, 2015, p. 294), in the proximal stimulus.

In the literature, a distinction is usually made between two types of perceptual completion, namely, between modal and amodal completions. This dichotomous classification goes back to Burke (1952), Glynn (1954), and Michotte and Burke (1951) and has influenced the research in the field for almost seven decades (see Wagemans, van Lier, & Scholl, 2006, for an entertaining essay on Michotte’s life, work and heritage). In their seminal work, Michotte, Thinès, and Crabbé (1964/1991) present a number of visual phenomena that evoke qualitatively different percepts of completeness and classify them as modal or amodal depending on their phenomenal appearance.

In essence, they call visual completions “modal” if the additions “present the same perceptual qualities (luminance and color) as the rest of the configuration” (p. 141), and otherwise “amodal.”^1^ In visual perception, modal completion includes, for example, “simple gap-filling” (p. 141) such as the filling of sensory holes in the blind spot or the connection of image fragments to a unified perceptual figure that is completely visible (as, e.g., in the stimuli investigated by Bobbit, 1942, which will be discussed later). In contrast to modal completion, amodal completion in visual perception is typically characterized by the connection of image fragments to a unified perceptual figure that appears partially (or temporarily) occluded, but it also includes the construction of perceptually self-occluding solids and the perceptual filling of “empty” objects (e.g., a rotating wire cube) with “something” (p. 161).

Although Michotte et al.'s usage of “modal” and “amodal completion” seems straightforward and intuitive at first sight, they do not offer a precise definition. More crucially, their examples of modal and amodal completions do not seem to be perfectly consistent, which makes it a bit difficult now to reconstruct definitions that might match their real notion.

**Figure 1.**
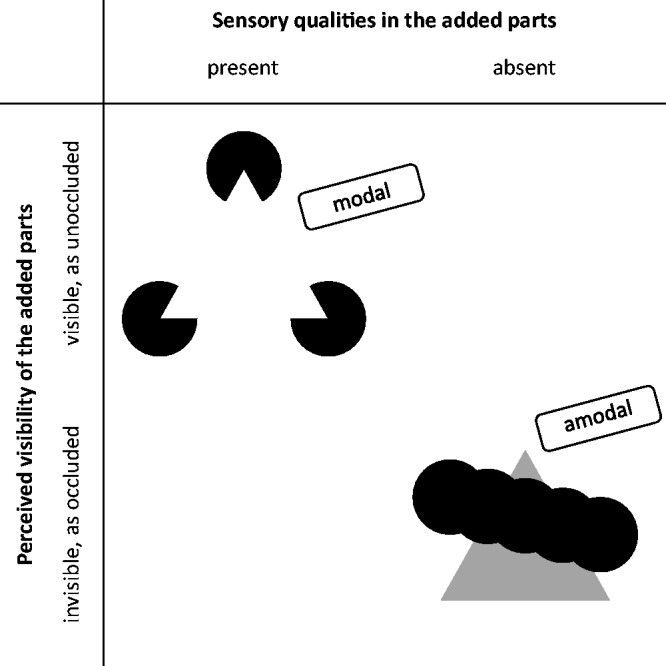
Two examples of today’s notion of modal and amodal completions: The famous white Kanizsa triangle (upper left, adapted from Kanizsa, 1979, Figure 12.1a) having margins without gradients on three black disks glows bright and white, which is why the completion is called “modal.” In contrast, no brightness or color is perceptible in the hidden but completed parts of the triangle in the lower right, which is why the completion is called “amodal.”

Nevertheless, especially promoted by Kanizsa, a clear and stable notion of modal and amodal completions has quickly emerged, which is widely accepted by the scientific community, even though it differs in a way from that of Michotte et al. Figure 1 shows two static examples of this common notion of surface completion in the domain of vision: a modally completed white “Kanizsa triangle” (Kanizsa, 1955/1987, 1979; cf. also Schumann, 1900, Figure 7) in the upper left and an amodally completed gray triangle in the lower right. “Amodal completion” typically refers to the perception of the *occluded parts* of objects, “using ‘amodal’ to refer to the *absence of sensory aspects*, e.g., brightness or color, in the parts of objects perceived to be behind other objects” (Kellman & Shipley, 1991, p. 143, our italics). That is, “amodal completion denotes the perception of parts of objects—the completed regions—that entirely lack visible attributes” (Pessoa et al., 1998, p. 728).

In contrast, “modal completion” typically refers to *unoccluded elements* and describes the situation when “perceived areas not delimited by physical differences appear *with sensory characteristics*” (Kellman & Shipley, 1991, pp. 143, 144, our italics). “The illusory^2^ contours and the central brightening [of the Kanizsa triangle] are modal in character: they are perceptually salient and appear to belong to the figure rather than the ground” (Pessoa et al., 1998, p. 728), It should be noted that the term “modal/amodal completion of an object” usually refers to the modal/amodal completion of the contour and surface of an object, which also implies the phenomenal impression of unity.

The distinction between modal and amodal completions is today (usually implicitly) based on the following two phenomenological criteria:

1. The *sensory-qualities criterion*. It is met if sensory aspects (e.g., brightness and color in vision) are present in the completed regions.
2. The *visibility criterion*. It is met if the completed region is visible, that is, not occluded.

Both criteria seem almost equivalent insofar as one criterion is usually met only when the other criterion is also met. If the criteria are met, the term “modal completion” is used, otherwise the term “amodal completion.” In a critical review of the current conception of perceptual completions, we will argue that the two criteria are actually not equivalent.

Both modal completion and amodal completion often involve an interpretation of occlusion: Amodal completion of a partly occluded object is typically induced by its *modally visible* parts, for example, by the gray regions in the lower right of Figure 1. In contrast, modal completion is typically induced by visible stimulus elements that, in the final percept, appear partly occluded *by* the modally completed object, for example, the Pacmen in the upper left of Figure 1, which are perceived as disks partly occluded by the triangle.

In this article, we discuss Michotte et al.’s (1964/1991) conception of modal and amodal completions and compare it with today’s notion. This is the basis on which we then argue that today’s dichotomy of modal and amodal completions is problematic in phenomenological, empirical, logical, and theoretical terms. Although this has of course no influence on the empirical findings regarding visual completions, it may, however, obstruct the view onto theoretical conclusions that could be drawn from them. This could in particular concern studies that directly address the question of which internal processes may cause modal and amodal completions (e.g., Anderson, 2007; Anderson, Singh, & Fleming, 2002; Carrigan, Palmer, & Kellman, 2016; Gerbino & Salmaso, 1985; Kellman, Garrigan, & Shipley, 2005; Kellman & Shipley, 1991; Kellman, Yin, & Shipley, 1998; Purghé & Coren, 1992; Ringach & Shapley, 1996; Shipley & Kellman, 1992; Singh, 2004; Tse, 1999a, 1999b; and many more). Finally, we present a more differentiated, theoretically neutral terminology that avoids the aforementioned problems and allows for a consistent phenomenological classification of completion phenomena.

For the sake of readability, we will sometimes only mention Michotte in the following sections. In these cases, we also refer implicitly to his collaborators on various publications.

## Michotte’s Versus Today’s Conception of Modal and Amodal Completions

In this section, we give a commented summary of the famous work of Michotte et al. (1964/1991) and show similarities and differences to today’s understanding of modal and amodal completions. We try to quote literally as often as possible because we want to present their thoughts as precisely as possible without unintentional misrepresentations. However, before doing so, we will briefly deal with the important distinction between the terms “completion” and “complement,” which was recently addressed by van Lier and Gerbino (2015).

### Perceptual Completion Versus Perceptual Complement

van Lier and Gerbino (2015, p. 294, footnote 2, original italics) notice that

[t]he French expression “compléments amodaux” […] has been occasionally translated into English as “amodal complements” […], but the prevalent contemporary usage is “amodal completion.” The difference between *complement* and *completion* points to the contrast between the phenomenological notion discussed by Michotte and Burke (1951) and the idea that amodal complements are the product of an active process of completion […].

Analogously, the expression “modal completion,” which should actually read as “modal complement” in most cases,

cannot be taken as denoting a hypothetical process of joining input fragments by means of illusory additions, according to what Kogo and Wagemans (2013) consider a common misinterpretation found in the literature on mid-level vision. Rather, it should be taken as denoting the phenomenal presence of parts devoid of an obvious local counterpart (a luminance difference, in the case of surface contours) but supported by global stimulus information and functional to the overall organization of the perceptual world. (van Lier & Gerbino, 2015, pp. 303, 304)

We consider it important to differentiate carefully between perceptual complements, whether given modally or amodally, and the hypothetically underlying completion processes. We will explicitly point out the difference by annotations in square brackets, also in literal quotations. Note that the translators T. R. Miles and E. Miles of Michotte et al. (1964/1991) are not the originators of the English translation “completion” of the French term “complements,” but they systematically translated “compléments” into “completion.”^3^

### Modal Complements

In the Introduction, Michotte et al. (1964/1991, pp. 140–142) characterize modal complements. Their key message is that modal complements possess phenomenal qualities such as—in the case of surfaces—brightness and color. This idea is still valid today. Michotte et al. assert that “no distinction is made by the subjects between the parts of the figures that were added and those that correspond to the system of stimuli.” They conclude that “[b]ecause these *additions present the same visual qualities (luminance^4^ and color) as the rest of the configuration*, we shall call this type of completion ‘modal’ completion [originally: nous appellerons ‘modal’ ce type de complément]” (p. 141, our italics). They point out that they use the term “modal” in the sense of Helmholtz “to describe the special qualitative character belonging to each sensory field” (footnote 6, p. 141). Note that this criterion resembles the sensory-qualities criterion formulated earlier, but it is stronger in that it also includes the rest of the configuration. This means that modal complements can only be constructed in the presence of *modal* counterparts of the same visual quality.

To illustrate their idea, they report a few examples that are “representative of the more simple type of perceptual completion [originally: compléments perceptifs],” which is “characterized by the fact that the subjects describe ‘better’ and simpler figures than those they were shown and that they make no mention of the gaps” (p. 141). The first examples involve filling phenomena: Filling of the blind spot and filling of tachistoscopically displayed figures observed in experiments with hemianopic subjects. Such phenomena are still representative of modal completion from today’s perspective.

**Figure 2.**
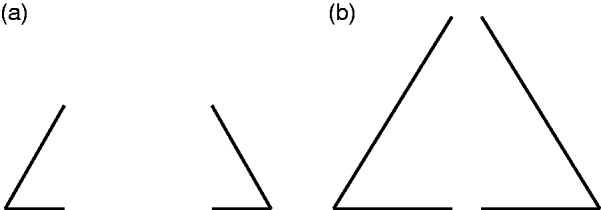
Stimuli used in Bobbit (1942) with less than 50% of the total perimeter of the triangle present (a) and more than 90% of the total perimeter of the triangle present (b). Adapted from Bobbit (1942, Figure I).

In contrast, the situation seems to be less clear for their next example: the perception of image fragments as a unified figure. They cite Bobbit (1942), who found that subjects judged two tachistoscopically displayed angles (Figure 2) as integrating into one triangle when more than 68% to 75% of the length of the perimeter were actually shown. However, Bobbit (1942) was actually interested in “the threshold for closure (defined as the point in the series representing the transition between the forms having the quality of twoness and those having the quality of oneness)” (p. 278) and defines “oneness” for his triangle stimuli such that “the two angles, while discriminably separate, were spontaneously perceived as being closely related, as belonging to one figure” (p. 278). Michotte et al. (1964/1991), however, maybe misdirected by the word “closure,” misquote Bobbit’s oneness criterion as seeing “a closed triangle [originally: un triangle fermé]” (p. 141), which has a completely different meaning. In our opinion, perceiving oneness in Bobbit’s sense does not in any way require modal completion. It is therefore hard for us to believe that the subjects really perceived the bridged gaps as modal complements, that is, as *indistinguishable* from the rest of the triangle, if the displayed perimeter exceeded a critical relative length (Figure 2(b)).

We suspect that Michotte et al. (1964/1991) would agree with us if they had noticed that their quote was inaccurate. They themselves point out later (in the section on amodal completion) with reference to their Figure 3.3 (p. 145), which shows a very similar “broken triangle” stimulus, that “from the very beginning there is an integration of the two parts of the figure, since what one sees is a single triangle whose contour is in two pieces.” They do not mention any modal complement but continue that “[a]s soon as a screen is put over the figure so as to cover the gaps, the contour,” there is “an amodal completion [originally: complément amodal] once again” (p. 145).

This shows that integration is not sufficient on its own in such a case to produce the completion [originally: complément]; other conditions have to be satisfied; in this case, those conditions make the line of demarcation seem to belong to the contour of the screen.^5^ (pp. 145, 146)

Summarized, they carefully distinguish between integration and completion/complements. *Complements require integration but not vice versa.*

**Figure 3.**
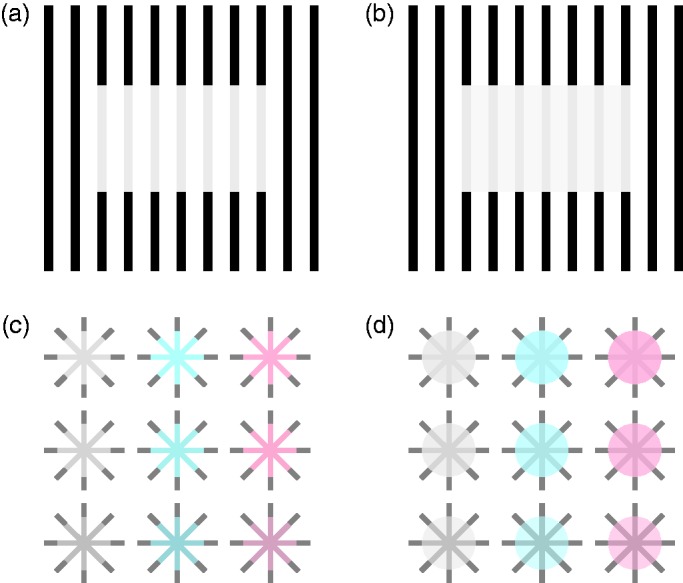
The distinction between apparent and perceptual transparency used in this article: “Apparent transparency” is present when the light gray bars in panel (a) appear to form a semitransparent rectangle floating in front of the black bars. The horizontal edges of the “rectangle” consist of both “real” *and* illusory contours, just like the Kanizsa triangle. Only three luminance levels are used here (black, white, and gray). In contrast, “perceptual transparency” requires physical discontinuities throughout (b). A fourth luminance level is therefore necessary so that the white background has been replaced with light gray bars in the area of the “rectangle.” (c) Apparent transparency of Ehrenstein figures in connection with neon color spreading. (d) Colored sectors were added that lead to perceptual transparency. Panel (a) adapted from White (1979, Figure 1).

Michotte et al. also discuss the “Rosenbach phenomenon,” which they classify as a case of apparent transparency, but which does not have much in common with the stimuli as shown in Figure 3. The phenomenon was first described by Rosenbach (1902) and later systematically investigated by Glynn (1954). It consists essentially in the observation that an element (e.g., a light gray rectangle against a white background) partially masked by a screen (e.g., a thin, opaque dark gray stripe) shines through the screen as if that screen were apparently transparent. This transparency effect only occurs under suitable luminance relations between element, screen, and background (Glynn, 1954, p. 138) and is supposed to be much stronger when the element is moved back and forth behind the screen (Rosenbach, 1902, p. 435). More critical, the effect “demands an adequate ‘set’ and certain period of training even under optimum physical conditions” (Glynn, 1954, p. 139) to occur at all.

“Rosenbach transparency,” if not imagery, would be clearly understood as modal completion of the partially masked element today because its surface complements have visual qualities such as brightness and color and are directly visible (albeit through a transparent layer). For Michotte et al. (1964/1991), however, it “form[s] an intermediate group between simple gap-filling and the completions we shall later term ‘amodal’ [originally: compléments ‘amodaux’]” (p. 141). They argue that “this gap-filling is not done by a simple addition of a missing part, but by the setting up of a structure of transparency, which necessarily involves a phenomenal duplication at the point of meeting” (p. 142). In this respect, Rosenbach transparency also has parallels to amodal complements under occlusion. Both views seem justified.

For the sake of clarity, we will use the terms “apparent transparency” and “perceptual transparency” in the following to distinguish between two different classes of stimuli, both of which evoke a transparency percept. With apparent transparency, we refer to fragmented stimuli that complement each other to form a semitransparent figure with partially illusory contour and surface, as shown in Figure 3(a) (“White’s illusion”; White, 1979) and (c) (neon color spreading; e.g., Bressan, Mingolla, Spillmann, & Watanabe, 1997). It is today clearly understood as modal completion (e.g., Nakayama, Shimojo, & Ramachandran, 1990). Although Michotte et al. do not mention it, the same line of argument as for Rosenbach transparency would also apply here. It is obvious that the transparency percept only occurs under certain luminance relations between the different stimulus elements.

More controversial is whether perceptual transparency (Figures 3(b) and (d); e.g., D’Zmura, Colantoni, Knoblauch, & Laget, 1997; Faul & Ekroll, 2011; Metelli, 1970; Singh & Anderson, 2002) can also be seen as a (modal) complement, since here, in contrast to apparent transparency, no illusory contour/surface is “added.”^6^ There is the idea that “notions like scission and layer decomposition, combined with grouping by surface colour similarity and contour good continuation satisfactorily account for perception [of transparency]” (Gerbino, 2015, p. 429). Therefore, one may find it inappropriate to speak of completion/complements when image regions are grouped in the absence of gaps. This seems to be in line with Michotte et al. (1964/1991, p. 140), who characterize modal complements as “certain details, certain elements contributing to the perceptual structure, which, unlike the others, do not correspond to any local stimulation.”

However, there may be good reasons to understand perceptual transparency as a modal complement: First, the transition between the seemingly dichotomous classes of apparent and perceptual transparency is continuous at both stimulus and perceptual levels (cf. in particular Figures 3(a) and (b)). Second, phenomenal duplication of a stimulus region (scission into transparent layer and background; Metzger, 1936) implies already that something is perceptually “added,” very similar to modal complements. This addition does not “correspond” to the local stimulation, which can easily be verified by isolating the stimulus region from its context. Third, understanding perceptual transparency as the result of a grouping process does not exclude that it is also a complement as the result of a completion process because completion may be a special form of grouping (cf. p. 27).

### Amodal Complements in Static Scenes

**Figure 4.**
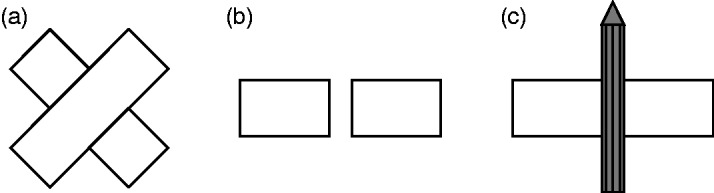
Static amodal complements (a, c). (a) Two squares separated by a gap, amodally completed to form a rectangle. (b) Two rectangles separated by a small gap. (c) The same rectangles as in panel (b), whose gap (and adjacent borders) is now “screened” with a pencil. The stimuli now complete amodally to one long rectangle, in front of which a pencil is placed (“screen effect”). Adapted from Michotte et al. (1964/1991, Figures 3.1 and 3.2).

After their overview of modal complements, Michotte et al. (1964/1991, pp. 142–149) present their concept of amodal complements in connection with the “simple screen effect” (Figure 4(a)), which is basically what is nowadays understood as amodal completion, because neither the sensory-qualities nor the visibility criterion is met.

Michotte et al. (1964/1991) explain that “[t]he essential feature of the screen effect is that *one object covers another without appearing to alter the integrity of the latter*” (p. 143, our italics). We assume that they use the term “screen effect” because certain stimuli radically change their perceptual character at the moment when a “screen” (e.g., a cardboard strip or a pencil) is placed in front of them (Figures 4(b) and (c)). “[C]ompletions [originally: compléments] that are ‘present’ in this manner should be termed ‘amodal’ to distinguish them from the previous kind” (p. 144). Note that “*the term amodal* has initially a purely negative significance, since it *indicates the absence of visual qualities (luminance and color) from the completion* of the figure [originally: complément figural]” (p. 144, our italics). While “the impression of the unity of the whole shape and the unbroken character of its contour is entirely compelling for the subjects” and “the shape, direction, and so on of the figural completion [original: complément figural] can be perfectly precise” (p. 144), the impression of the continuity of color is less compelling. “It seems rather to become established secondarily, by reason of the unity of the whole shape, as an overall property of it” (p. 144). Finally, they distinguish amodal completion, which is stimulus-driven, from imagination, “which implies the presence of sensory qualities” (p. 146).

**Figure 5.**
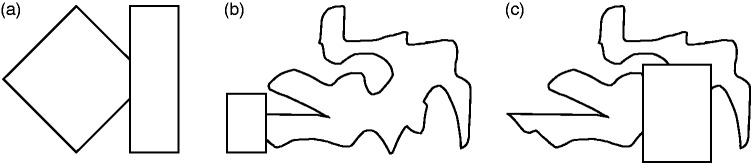
Amodal complements can even appear if only one side of a figure is occluded (a), but they also appear with irregular, unfamiliar shapes (b), whereby the character of the complements is strongly affected by the specific occlusion cues (c). Panel (a) adapted from Michotte et al. (1964/1991, Figure 3.3).

**Figure 6.**
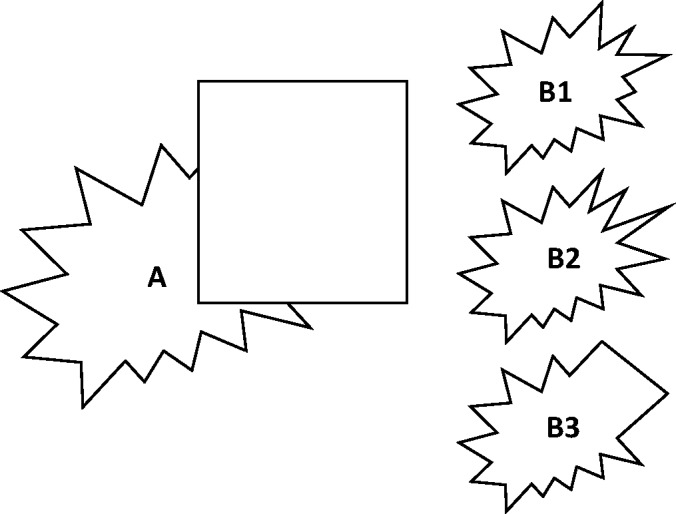
Fuzzy completions/complements. Pattern A comprises a random shape partly occluded by a rectangle. Locally viewed, B1, B2, and B3 all seem to offer plausible completions of A. However, only the completed parts of B1 and B2 are in accordance with the visible edges and angles of A, which is why the completions B1 and B2 do not seem unlikely. In contrast, the completed parts of B3 have clearly different edge lengths and angles, which is why the completion B3 seems unlikely. Adapted from van Lier (1999, Figure 3).

While all of the aforementioned is consistent with today’s view, two considerations need to be discussed in more detail:

First, Michotte et al. (1964/1991) note that *amodal completion occurs even when only one side of a figure is covered by a screen*, which is undoubtedly true (Figure 5(a)), and they suppose that “[t]his effect occurs in particular when the figure has ‘pregnance’, [originally: une forme prégnante] as in the case of a square, a rhombus, or a circle” (p. 146), which is certainly not wrong either. We do not, however, agree to their idea that “in this case the completion is often carried out by the imagination, which implies the presence of sensory qualities and, therefore, invalidates the example” (p. 146). The literature is full of countless examples that show that absolutely *every* element perceived as occluded is in some way amodally completed. Figure 5(b) shows, for example, an irregular, unfamiliar contour with a rectangle in front of it. It is obviously completed amodally, with a “good continuation” of the part adjacent to the occluder (cf. e.g., Kanizsa, 1970; Kellman & Shipley, 1991; but also e.g., Tse, 1999a, 1999b; van Lier, van der Helm, & Leeuwenberg, 1995). The same applies to the contour in Figure 5(c). Here, the rectangular occluder is placed at a different position, which makes the percept more ambiguous. A similar (but different) shape as in Figure 5(b) may appear or two amodal complements of two separate objects: one corresponding to the contour to the left of the rectangle and one corresponding to the remaining contour. Whichever percept emerges, it appears immediately and involuntarily, even if one *knows* what the contour actually looks like, or more precisely, what it *might* look like, namely, as shown in Figure 5(b). It can therefore be said that the outcome is clearly amodal and perceptual without significant imaginary influences.^7^ Note that more global and more abstract surface/object properties can also influence amodal completion (e.g., in accordance with so-called fuzzy regularities of visible surface/object parts as shown in Figure 6; cf. van Lier, 1999).

**Figure 7.**
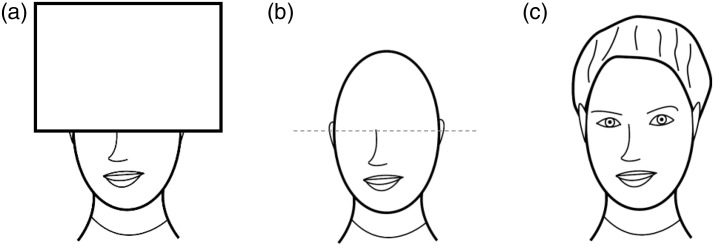
A stimulus resembling a partially occluded face (a) and a possible resulting amodal percept (b). Imaginary additions may also contain configurational elements such as eyes, eyebrows, and so on as well as surface features such as skin color (c).

Second, Michotte et al. (1964/1991) suggest that “acquired experience can have an analogous effect” to amodal completion. For example, a partly occluded human head “clearly continues” behind an occluder, “but its shape is indeterminate” (p. 146) and “these prolongations remain imprecise. Because of their influence, however, the uncovered parts are sufficient to identify the nature of the objects and so make adaptation to the environment possible” (p. 147). In our opinion, this is anything but analogous to amodal completion. For example, as illustrated in Figure 7, the visible parts of a partially occluded face can evoke the clear, knowledge-based *imagination* that certain configurational elements and features are missing in certain places, but *it is in the nature of amodal complements that they do not contain visible surface features* such as brightness, color, or texture (Davis & Driver, 1998, p. 753; Pessoa et al., 1998, p. 731). The exact course of the completed contour and thus the exact extent of the completed surface depends of course on the specific position of the occluder (Tse, 1999b), but the visual system should always find a stable, albeit perhaps a vague amodal solution (cf. van Lier, 1999).

Due to the partially contradictory statements in their publication, it is difficult to reconstruct the real position of Michotte at al. (1964/1991) in every detail, but their conclusion is that the presence of amodal complements “is governed by the laws of organization of the perceptual field,” which “excludes any attempt at an explanation based on inferences or on the intervention of mental images” (pp. 164, 165).

Michotte et al. (1964/1991) complete the section on amodal complements in static scenes with thoughts on the perception of (three-dimensional) solids and self-occlusion. They discuss the example of a sphere, from which only one hemisphere is visible from every point of view, but which is nevertheless perceived as a complete sphere or ball, because

[t]he edge of the presented hemisphere does not in the experimental conditions act as a limit to the hemisphere’s surface, and since at this point there is no limit to this surface in the front to back direction, it necessarily seems to be continued in this direction. (p. 148)

This is consistent with today’s view (e.g., Ekroll, Sayim, & Wagemans, 2013, 2017). We think that especially Tse (1999a, 1999b)—regarding mergeability and other factors of amodal volume completion—and van Lier and Wagemans (1999)—regarding the influence of structural object aspects on amodal object completion—present interesting thoughts and insights on various questions posed by Michotte et al. in this context.

### Amodal Complements in Dynamic Scenes

Michotte et al. (1964/1991, pp. 149–161) also discuss related dynamic phenomena. They review experiments in which a moving (or stationary) target object appears to be gradually covered and uncovered by a stationary (or moving) occluder. The fundamental observation is that the target object continues to exist throughout and retains its perceived shape, even if it is not visible at all for a short time. Summarized, these “most simple cases” (Michotte et al., 1964/1991, p. 150) of dynamic covering and uncovering show “the permanence, in amodal form, of objects completely hidden” (Michotte et al., 1964/1991, p. 149), that is, that *amodal complements do not require continuous stimulation by visible inducers.* Instead, the life span of perceptual objects can also be extended amodally if (temporarily) no stimulation occurs at all (e.g., also during blank interstimulus intervals (ISIs); Scherzer & Ekroll, 2009). This is consistent with today’s view.

Michotte et al. also report similar observations when the occluder was indistinguishable from the background, that is, invisible to the subjects. The only difference was that the moving target now seemed to gradually disappear in (or reappear from) a modal illusory stationary slit in the background or that the stationary target was gradually covered and uncovered by a modal illusory moving occluder. From our point of view, these observations show that also *the presence of adequate spatiotemporal cues can induce illusory occluders that operate in the same way as “real” occluders*, that is, the occluders induce or coincide with the amodal complement of the target.^8^

**Figure 8.**
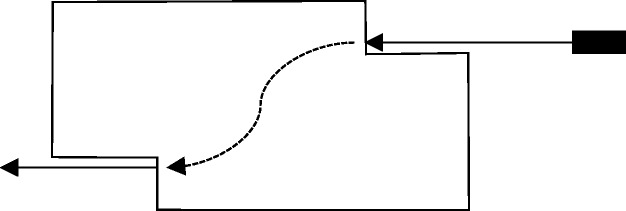
The tunnel effect. A continuously moving object (upper right) disappears behind a screen and reappears shortly afterwards (lower left). Under appropriate conditions, the object is perceived as moving continuously behind the screen (dashed line). Adapted from Burke (1952, Figure 3).

Another interesting phenomenon of “amodal persistence” is the “tunnel effect” (e.g., Burke, 1952; Sampaio, 1943). There are several variations of it, but in a basic variant a target element in continuous motion disappears behind a screen. After, for example, 1 second an identical looking target element appears behind the screen at the same or at a different position (Figure 8). Two important observations can then be made under appropriate conditions: First, “the moving object goes behind the tunnel” (Michotte et al., 1964/1991, p. 152). This again shows that *perceptual objects can continue to exist amodally for a while without visible inducers*.^9^ Second, “in spite of the presence of the screen, the movement is continuous and uniform” and “[i]t seems that *there is no difference between a movement of this kind and a movement exposed throughout*” (p. 152, our italics). The motion path in the tunnel is interpolated in a smooth, “natural” way (e.g., S-shaped as shown in Figure 8), even if the two visible motion paths do not lie on the same straight line.

According to Michotte et al., the tunnel effect is also related to the Phi phenomenon, that is, “pure movement” (“reine Bewegung”; Wertheimer, 1912) or “shadow movement,” which is detached from any carrier object. However, they also acknowledge that “[i]n other respects […] the two phenomena are quite different, particularly because of the existence in the tunnel effect of the exposed parts of the movement, which influence the apparent speed of the moving object during the hidden phase” (Michotte et al., 1964/1991, p. 153).

Note that the tunnel effect is very robust, as it also occurs with static stimuli (Wertheimer, 1912). Scherzer and Ekroll (2009, 2012) found that objects performing a Beta movement (apparent motion) seem to move continuously behind a screen under appropriate conditions. Beta movement is the perception of a moving object despite discontinuous stimulation, that is, the successive presentation of still images in which the target stimulus is displaced in discrete steps (cf. Steinman, Pizlo, & Pizlo, 2000).

### Amodal Complements Without Cover

It is surprising that some thoughts of Michotte et al. (1964/1991) about “amodal completion [originally: complément amodal] without cover” (pp. 161–163) have apparently never been taken up by the scientific community. Otherwise, it would have become clear that the authors interpreted “compléments amodaux” in a much broader sense than is the case today, where it is closely linked to occlusion, especially in the context of surface completion.

They give the example of a rotating “Necker cube” (Necker, 1832, p. 336) made of wire, which is described by their subjects as “moving, as if it were not just a mere skeleton but a receptacle filled with ‘something’ that appears to be enclosed in the cube and is moved with it” (Michotte et al., 1964/1991, p. 161).^10^ For Michotte et al., the cube “seems completely transparent and consequently has no modal character” (p. 161), especially when a structured background is visible through the (mass of the) cube. It is therefore considered amodal.

Michotte et al. also give stereoscopic and stereokinetic examples of outline figures in which surface and depth interpolation occurs, leading to the “amodal” perception of transparent objects with rigid walls.

All these examples show that *for Michotte et al., amodal perception is not limited to cases of occlusion*. However, applying the two criteria that, in our opinion, best characterize today’s view, the examples would clearly fall into the category of modal completion, as the transparent indefinable substance contains material qualities (sensory-qualities criterion) and is not occluded, that is, directly visible (visibility criterion).^11^

### Summary

The conception of modal and amodal complements presented by Michotte et al. (1964/1991) does not always appear to us to be completely consistent on the basis of the examples and explanations given, but it certainly differs in parts from today’s view. This is mainly because Michotte et al. characterize modal complements by the fact that “these additions present the same visual qualities (luminance and color) as the rest of the configuration“ (p. 141). With the exception of apparent transparency, which forms an intermediate group, Michotte et al. consider all complements that do not meet the aforementioned criterion as amodal, regardless of whether they are visible or invisible/hidden.

Today, modal and amodal complements are (usually implicitly) understood as dichotomous categories. Unlike Michotte et al., however, the classification into one of the two categories is strictly based on the sensory-qualities und the visibility criterion. Moreover, there is now a consensus that modal and especially amodal complements are not a product of imagination but are constructed according to fixed perceptual regularities. Compared with Michotte et al., some differences and extensions in today’s classification of certain phenomena can be observed:

1. According to Michotte et al., Rosenbach transparency forms an intermediate group between simple gap-filling and amodal complements. Although not mentioned by the authors, the same consideration would probably also apply to apparent and perceptual transparency (Figure 3). Today, Rosenbach transparency and apparent transparency would be considered a modal complement, as it contains visual qualities (sensory-qualities criterion) and refers to directly visible regions (visibility criterion). It is controversial whether perceptual transparency can also be understood as a modal complement (completion) or not as a complement (completion) at all.
2. For Michotte et al. (1964/1991), the complement of a one-sided occluded figure is “likely” to be “often carried out by the imagination” (p. 146) when the figure has “pregnance” (like a square, a rhombus, or a circle). This is inconsistent with today’s view that amodal complements of partly occluded figures are perceptual and not imaginative, although it is clearly influenced by top-down processes.
3. While Michotte et al. focused on visual complements of objects and the interpolation of motion under occlusion, the concept of perceptual complements/completion has been extended to texture and motion spreading (Watanabe & Cavanagh, 1991) and the completion of depth (Nakayama & Shimojo, 1990), for example, which are today understood as modal completion (Pessoa et al., 1998, p. 732). The occurrence of amodal complements/completion has also been shown in nonvisual domains, for example, in auditory scene analysis (Bregman, 1981). Furthermore, ethological studies show that completion also occurs in animals (e.g., Bakin, Nakayama, & Gilbert, 2000; Lee & Nguyen, 2001; Nieder, 2002; Sovrano & Bisazza, 2008, 2009).
4. Complements without cover, which Michotte et al. considered as amodal, would probably have to be considered as modal today if the sensory-qualities and the visibility criterion, which are both met in this case, were applied.

## Problems With Michotte’s Conception of Modal and Amodal Percepts

Taking Michotte et al. literally, visual percepts can be classified either as modal, amodal, or apparently transparent (as an intermediate group). This trichotome division appears suitable for figures and surfaces whose complements are characterized by the presence or absence of visual qualities such as brightness and color.

To apply this categorization principle to the perception of motion, its visual qualities would have to be identified, as the visual qualities of objects such as brightness and color are not defined for motion. Michotte et al. implicitly mention continuity and uniformity as visual qualities of motion. Thus, analogous to the categorization of perceptual complements of objects, motion would have to be considered modal if it is continuous and uniform, and otherwise amodal.

In our opinion, however, both the trichotome classification of object percepts and the dichotome classification of motion percepts by Michotte et al. are problematic for different reasons, as we will briefly explain in the following subsections.

### Visual Complements of Figures and Surfaces

Taking the strict definition of modal complements of Michotte et al. as a basis and considering perceived transparency (Figure 3) as a different class of visual complements, amodal complements form a natural residual class. If stimulus-driven perceptual outcomes are clearly distinguished from imagination, all visual complements of figures and surfaces could in principle easily be assigned to one of the three categories mentioned.

In our opinion, however, this division is problematic for two reasons: First, the residual class of amodal complements is so large that it contains many phenomena that have neither phenomenally nor structurally significant similarities (e.g., the screen effect on the one hand and complements without cover such as the rotating Necker cube on the other). It is therefore highly questionable whether it makes sense from a theoretical point of view to classify these different phenomena in a common category.

Second, the restriction of the definition of *modal* complements only to the locally specified surface attributes brightness and color and thus the exclusion of globally specified object attributes such as unity, shape/contour, or surface material/quality, seems unjustified from a phenomenological point of view: Scherzer and Ekroll (2015, p. 11) suppose that “[t]he notion that luminance and color are visual qualities while contour and shape are not may be motivated through reference to ideas from sensory physiology.” They argue that, “[i]n terms of immediate visual experience, it would seem reasonable to regard contour and shape as visual qualities on par with luminance and color.” The unity, shape, and contour of an amodally completed figure, for example, are often visually just as evident and salient as the brightness or color of a directly visible surface. Thus, unity, shape, and contour establish the (visual) modality in much the same way as brightness and color.^12^ If, however, such globally specified object attributes were understood as visual qualities just like the locally specified ones, then even “typically amodal” complements of partially occluded surfaces (as in the screen effect) would have to be regarded as modal, at least with regard to certain globally specified attributes (cf. Scherzer & Ekroll, 2015, pp. 11, 12). Then, the trichotomy of visual complements proposed by Michotte et al. (1964/1991) could no longer be maintained.

### Visual Complements of Motion

Burke as well as Michotte et al. (1964/1991) interpret the tunnel effect as *amodal* completion of motion, or “amodal completion of kinematic structures [originally: complément amodal des structures cinétiques]” (p. 156). However, there is a contradiction here because “in spite of the presence of the screen, the movement is continuous and uniform” and “[i]t seems that there is no difference between a movement of this kind and a movement exposed throughout” (Burke, 1952, p. 152). This corresponds, applied to motion, exactly to the criterion for *modal* complements, namely, that “the additions present the same visual qualities as the rest of the configuration” (Michotte et al., 1964/1991, p. 141). It seems as if they tacitly apply the visibility criterion here, but not with reference to the movement, but instead to its carrier object. This seems strange, particularly as they did not use this criterion for objects in any other context.

Although Michotte et al. carefully distinguish between the “strictly kinematic aspects of the tunnel effect” and “question[s] about the object that performs the movement” (p. 156), the seeming contradiction between the modal perception of movement on the one hand and the amodal appearance of the object on the other is not addressed. It therefore remains unclear why the tunnel effect, which is significantly characterized by the movement of a temporarily hidden object, should be understood as (purely) amodal phenomenon.

**Figure 9.**
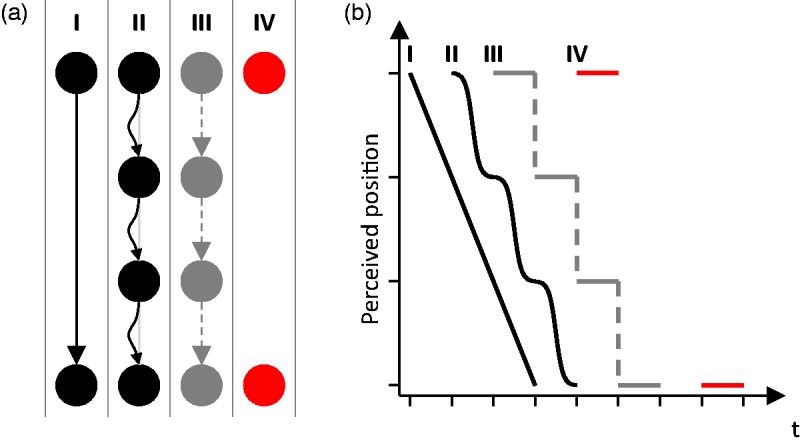
(a) Sketch of four possible percepts of different sampled motion stimuli (Scherzer & Ekroll, 2009, 2012): continuous linear motion (I), continuous accelerated/decelerated motion (II), jerky motion (III) with abrupt jumps (dashed), and appearance/disappearance of two different static objects in different places at different times (IV). The corresponding perceived positions/motion paths over time are depicted in panel (b) (for the sake of clarity, the four graphs were plotted next to each other instead of overlapping each other). Note the visible gaps (dashed) between successive positions in percept III and the missing motion in percept IV.

Therefore, we disagree with Michotte et al. on this point and prefer to classify the motion impression in the tunnel effect as modal. Motion continuity and uniformity can be summarized as motion smoothness, which is probably the most important visual quality of motion and which is closely related to the (change in) speed of movement over time. Scherzer and Ekroll (2009, 2012) empirically showed that even in apparent motion behind an occluding screen the motion path is interpolated under suitable conditions in such a way that a smooth, continuous, “real” impression of motion is produced (percepts I and II in Figure 9). It therefore seems natural that in the tunnel effect only the temporarily hidden carrier object should be understood as amodally continued over space and time, in exactly the same way as a temporarily occluded stationary object is continued amodally. In contrast, the smooth motion interpolation of a temporarily hidden carrier object should be considered a *modal* complement of the visible motion trajectories.

**Figure 10.**
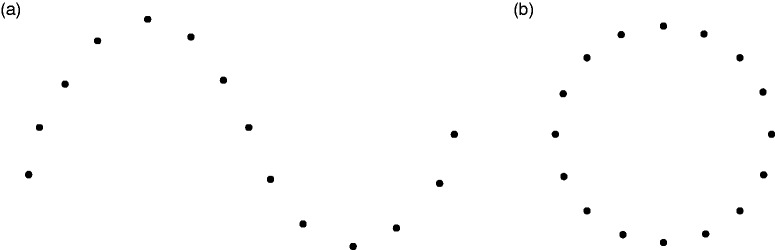
“Bridge lines” (Metzger, 1936), also called “virtual lines” (Kanizsa, 1955/1987). A sine-like wave (a) and a circle-like shape (b). Adapted from Kanizsa (1955/1987, Figures 4.1a and 4.3).

Conversely, a jerky “displacement percept” with abrupt jumps (percept III in Figure 9), as it occurs under certain conditions in apparent motion (Scherzer & Ekroll, 2009, 2012), cannot be understood as modal, as it is qualitatively different from a “real” motion percept and lacks smoothness as the most important visual quality of motion. In this respect, it is analogous to an amodally completed surface whose added parts are qualitatively different from the visible ones and lack the most important visual attributes of surfaces, namely, brightness and color. However, in contrast to amodal complements, the trace of the perceived object positions contains visible gaps. Therefore, percept III seems to be best regarded as analogous to the result of the integration of visibly disconnected elements (Michotte et al., 1964/1991, pp. 145, 146) rather than analogous to amodal complements. Either way, it is qualitatively completely different from the modal motion percepts I and II (and from the static percept IV).

## Problems With Today’s Conception of Modal and Amodal Percepts

As already discussed in the previous sections, the current concept of modal and amodal perceptions considerably differs from that of Michotte et al. But even today’s dichotomy is problematic in phenomenological, empirical, logical, and theoretical terms, mainly as the distinction is made on the basis of the sensory-qualities and visibility criterion (see Introduction section and Figure 1), which are highly, but not totally correlated. We will demonstrate this by presenting some examples in the following two subsections, which each only meet exactly one criterion and therefore cannot be classified consistently.

### Amodal Percepts Without Occlusion

Michotte et al. (1964/1991, pp. 161–163) present various examples of “amodal completion [originally: complément amodal] without cover,” which, however, meet both the sensory-qualities and the visibility criterion and are therefore regarded today as modal percepts.

Yet there are also examples of today’s amodal percepts without occlusion that do not meet the sensory-qualities criterion, even though they meet the visibility criterion. A class of completion phenomena to which this applies are so-called bridge lines (“Brückenlinien,” Metzger, 1936) or “virtual lines” (“linee virtuali,” Kanizsa, 1955/1987). This is how collections of dots are called, which are visually connected to a clearly defined contour in a regular manner (Figure 10(a)). The phenomenal impression of the figure resembles an amodal rather than a modal percept (“amodal character”; Kanizsa, 1955/1987, p. 44) but without any influence of occlusion.^13^ If the contour is perceived as closed (Figure 10(b)), even the perception of a flat form, for example, a disk, can arise. Its surface, however, then does not contain any visual qualities such as brightness or color. It appears virtually amodal but not occluded. See Scherzer and Ekroll (2015, pp. 12, 13) for more thoughts on this.

**Figure 11.**
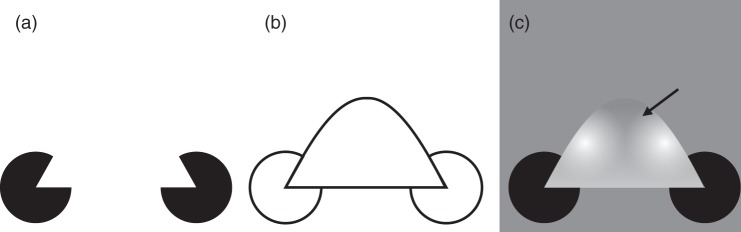
An “incomplete” illusory (Kanizsa) triangle producing a “fuzzy figure” (a). Its contour and surface become increasingly diffuse as the distance from the inducing elements increases. (b) A conceivable percept in a schematic manner. Panel (c) illustrates a possible “brightening map,” where white represents strong brightening and grayer tones a rather diffuse impression in which the surface seems to merge continuously with the physically identical background (here gray for illustration purposes). The arrow points to the transition area between brightened regions and the fusion of the surface with the background.

Another class of completion phenomena that do not fully meet the sensory-qualities criterion, although they meet the visibility criterion, are Kanizsa-like figures with a large distance among the inducing elements as well as “incomplete” Kanizsa-like figures as in Figure 11(a). The special characteristic of such “fuzzy figures” is that their contours become more diffuse with increasing distance from the inducers. A conceivable percept of the contour is shown schematically in Figure 11(b). The completion of contours seems to be carried out in a regular manner, that is, it always occurs in the same way for a specific stimulus. However, the clarity of the contour decreases significantly with increasing spatial distance from the inducers and the uncertainty about its shape increases. Also, the phenomenal impression is not constant across the entire surface. This is illustrated in a possible “brightening map” in Figure 11(c): As the clarity of the contour decreases with increasing distance from the inducers, the brightening of the surface also decreases until it seems to fuse with the background (shown here in gray for illustration).

**Figure 12.**
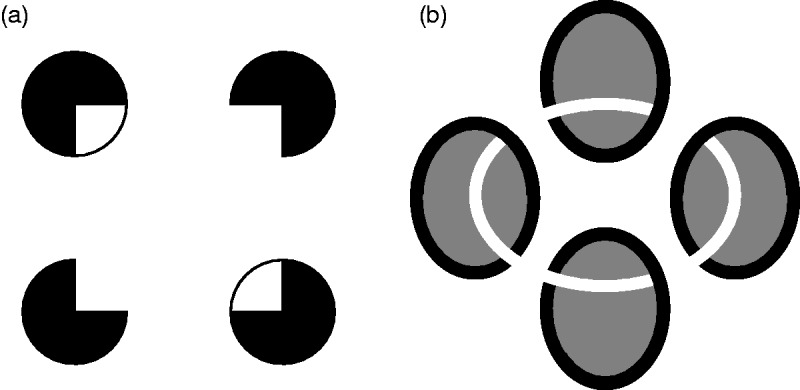
Quasimodal completion of the white square (a) and quasimodal completion of the white ring with depth impression (b). In quasimodal displays, modally and amodally completed contours join. Adapted from Kellman et al. (1998, Figures 4 and 5).

While the surface impression near the inducers is clearly modal, the presence of visual attributes such as brightness (or color) decreases continuously with increasing distance, until finally no difference to the background is discernible anymore. The completion of the surface can therefore in its entirety neither be called modal nor amodal, as the type of complement obviously depends on the location on the surface. This also applies in a similar way to quasimodal percepts (Figure 12; Kellman et al., 1998).

The consequences of these observations and the problem of determining an exact distinction between modal and amodal regions will be discussed further later.

### Modal Percepts Despite Occlusion

There exist not only completion phenomena that evoke an amodal percept despite direct visibility but also reverse cases in which a modal percept appears despite occlusion, that is, that the sensory-qualities criterion is met, while the visibility criterion is not.

**Figure 13.**
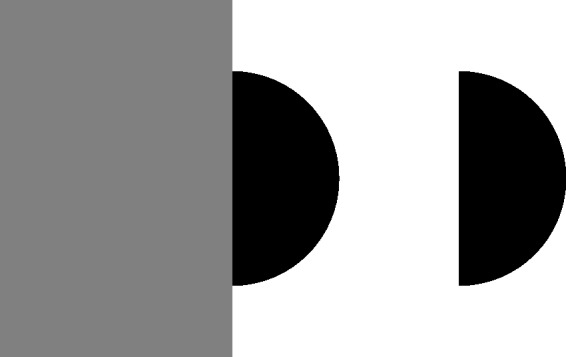
The occlusion illusion. The two black semicircles are physically identical, but the one adjacent to an occluder looks considerably larger than the one standing alone. Adapted from Palmer et al. (2007, Figure 1d).

Evidence of the existence of such phenomena is provided by the occlusion illusion (Palmer, 1999), which consists in the observation that a stimulus element appears larger when it is adjacent to an occluder than when it is presented in isolation. This observation originally goes back to Kanizsa and Luccio (1978; cf. also Vezzani, 1999).^14^ As demonstrated in Figure 13 and experimentally shown by Palmer, Brooks, and Lai (2007) and Palmer and Schloss (2009), the semicircle on the left appears not only as a full circle by the addition of an amodal complement behind the occluding rectangle but also as if slightly more than half of the semicircle were uncovered, that is, directly visible. Because the surface of this small additional stripe contains visual qualities (here brightness), Palmer et al. (2007) use the term “partial modal completion.”

Up to this point one could expect a size or shape illusion, as there are many in the literature, but it is particularly interesting to ask how the additional space for the modally completed stripe was released. An obvious possibility would be a slight displacement of one or both elements, but Palmer et al. (2007, p. 669, our italics) speculate “that the visual system somehow manages to see the partly occluded object as spatially extended *perpendicular* to the occluding edge without perceiving any difference in the positions of the regions attached to the edge.” This would, however, imply that a modal impression would be possible despite occlusion, which would lead to the paradoxical situation that this region would appear visible and yet occluded at the same time.

**Figure 14.**
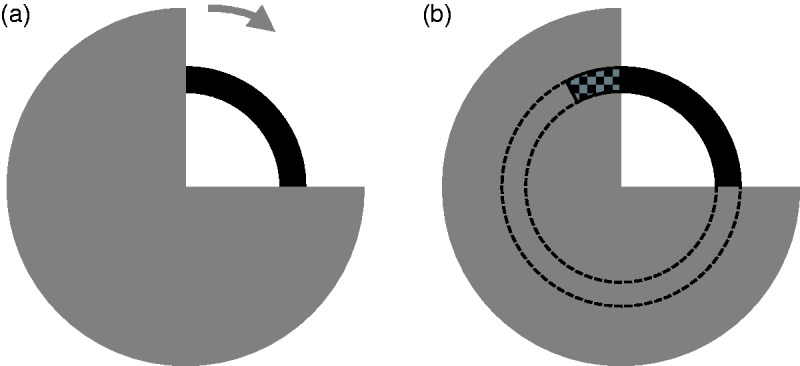
Schematic representation of the visibility paradox (Scherzer & Ekroll, 2015). A rotating disk sector in front of a stationary full ring (a) leads under suitable conditions not only to an amodal ring complement (dashed in b) but also to a partial *modal* complement that “overlaps” the rotating disk sector *without occluding it* (checkboard pattern in b). The visibility paradox is that both the modal ring complement and the rotating disk sector appear to be visible simultaneously at the same location in the visual field, that is, the perceived angle of the modal visible ring sector and the perceived angle of the occluding disk sector add up to more than 360°.

Scherzer und Ekroll (2015) showed in experiments with comparable dynamic stimuli (Figure 14), in which a circular arrangement prevented the elements from evading, that by partial modal completion in certain regions of the visual field not only the foreground (a disk sector) appears directly visible and with visual qualities (brightness and color), that is, modal, but also the background (a ring) that is actually occluded there. In some conditions, the effect was even greater than in the static occlusion illusion. The speculation of Palmer et al. (2007) seems to have been confirmed.

The visible character of the partially modally completed region is clearly inconsistent with any occlusion interpretation, which is why we might not be *aware* of it (especially in static situations as shown in Figure 13). However, this does not exclude that the occlusion of this region may be represented internally, that is, that it is actually *perceived*. This seemingly inconsistent situation could be resolved by assuming that the underlying visual processes operate on complex representations of different depth layers (e.g., Grossberg, 1994; Kogo, Strecha, van Gool, & Wagemans, 2010). Scherzer and Ekroll’s theoretical explanation for this “visibility paradox” can be found in Scherzer and Ekroll (2015, p. 10).

These examples of partial modal completion despite occlusion in static and dynamic scenes refer to the visual qualities of surfaces (brightness and color), but modal percepts despite occlusion are also possible in motion perception, as discussed earlier in the context of the tunnel effect: The motion interpolation behind a masking screen should be considered modal, with the modal complement referring to the (visual quality of the) smoothness of the interpolation, if under appropriate conditions it is not phenomenally discernible from real motion.

## Proposal for Alternative Classification Criteria

**Figure 15.**
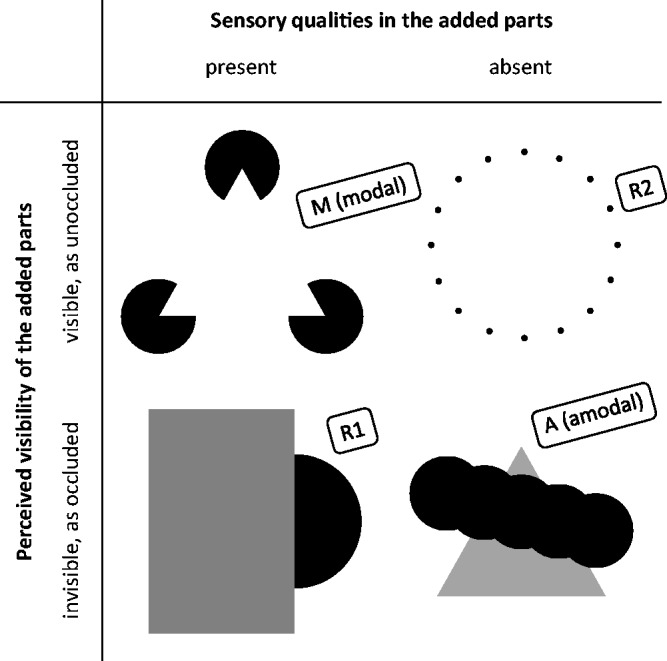
While Kanizsa-like figures (upper left) and the added parts of partly occluded objects (lower right) are typically categorized as modal and amodal complements, respectively, phenomena such as the occlusion illusion (lower left) and virtual lines/figures (upper right) fall into residual complement classes R1 and R2, respectively, which shows that the dichotomous division into modal and amodal complements is inappropriate. Note that the stimulus in the lower left leads to two phenomena: a “conventional” amodal complement and a partial modal complement (“occlusion illusion”; Palmer et al., 2007). The latter phenomenon is the reason why we assign the stimulus to the class R1 here.

The phenomena presented earlier show that the dichotomy of modal and amodal complements is not suitable in its current form, as in certain cases only the sensory-qualities or only the visibility criterion is met (Figure 15). In these cases, no consistent classification of the visual complement as modal or amodal is possible. In this section, we discuss several possible extensions of the existing scheme and finally propose an alternative classification of visual completion phenomena (and visual percepts in general) that seems consistent with all known empirical findings.

### Attempt 1: Both Sensory-Qualities and Visibility Criterion

Probably, the easiest way to solve the problem that in some cases only one of the two criteria applies would be to classify only those phenomena as modal (type M) that meet both criteria and those phenomena as amodal (type A) that do not meet either criterion. For the two residual classes of phenomena, which each meet exactly one of the two criteria, there would have to be a third and fourth type accordingly (R1 and R2, respectively; Figure 15).^15^

The drawback of this solution is that it ignores essential phenomenological and presumably structural similarities that exist between the four categories, for example

- the presence of visual qualities both in the Kanizsa triangle (type M) and in the “partially modally” completed regions in the occlusion illusion (type R1),
- the direct visibility of both the Kanizsa triangle (type M) and virtual figures (type R2),
- the perception of occlusion both in the “amodally” and in the “partially modally” completed regions in the occlusion illusion (type A and R1, respectively) and
- the missing visual qualities of both amodal complements (type A) and virtual figures (type R2).
- We therefore consider this approach inappropriate.

### Attempt 2: Visibility Criterion Only

Another obvious solution would be to use only the visibility criterion, whereby a clear assignment to one of the two dichotomous categories would presumably always be ensured. From an empirical point of view, however, this is problematic because the perception of direct visibility is possible despite the impression of occlusion (occlusion illusion; Figures 13 and 14) and probably occurs (unnoticed) more or less clearly in almost all stimulus situations, that is, this is by no means a rare limiting case. Therefore, this approach appears inconsistent, as perceived direct visibility cannot be equated with perceived “nonocclusion,” which is a prerequisite of the visibility criterion.

One could weaken the criterion in the sense that only the perceived (in)visibility could be used to categorize completion phenomena, but this would theoretically be unmotivated and furthermore any reference to the pervasive perceptual concept of occlusion and completion/continuation would be lost. From a theoretical point of view, however, such a reference seems indispensable for a meaningful classification of completion phenomena. Moreover, the objection that there are phenomenological and structural similarities between completion phenomena that are not captured by the classification scheme applies in part also to a scheme based on the weakened visibility criterion.

If the perceived (non)occlusion instead of perceived (in)visibility was used as an alternative criterion, similar objections would apply.

### Attempt 3: Sensory-Qualities Criterion Only

Conversely, the idea could be to use only the sensory-qualities criterion to classify completions, which would supposedly always ensure a clear assignment to one of the two dichotomous categories. With some corrections, as outlined earlier, this would probably also largely correspond to the view of Michotte et al.

However, we have already argued earlier that from a theoretical and phenomenological point of view this criterion appears suboptimal because the amodal residual class would be very large and also phenomenally heterogeneous. Accordingly, a further subdivision into suitable units would be necessary for a systematic analysis.

Another problem is that for some completion phenomena the sensory-qualities criterion only applies in certain regions of the complement and not in others (Figures 11 to 14). It follows that a phenomenologically precise classification cannot be achieved at a global level but must properly consider local differences, for example, at the level of “subcomplements.”

In addition, the transition between the presence and absence of sensory qualities can be continuous (see especially Figures 11 and 12) so that it would be necessary to specify a threshold value for the “amount of presence” at which the sensory qualities required by the criterion are regarded as fulfilled. This clearly shows that the dichotomy of modal and amodal completions (or complements) is generally inadequate in its current form.

### Alternative Classification Scheme for Visual Percepts

In this subsection, we present a proposal that solves these problems.

#### The conclusive-sensory-evidence hypothesis

Scherzer and Ekroll (2015) proposed a theoretically neutral terminology, which seems consistent with all known empirical findings and phenomenological observations and allows a consistent classification of completion phenomena. The proposal will be summarized and clarified in some places in this section.

Scherzer and Ekroll (2015) hypothesize that “visual percepts are experienced as modal whenever they are based on sufficiently conclusive sensory evidence for particular attributes of the visual scene and as amodal otherwise” (p. 11). They point out that the idea that phenomenal visibility represents the conclusiveness of the underlying sensory evidence is similar to previous suggestions linking qualia to the reliability of perceptual inferences (Gregory, 1997; Hibbard, 2008; Ramachandran & Hirstein, 1997). Sensory evidence can be seen as related to what Rock (1983, pp. 120–125) called stimulus support, but it is not the same (see the corresponding subsection on this later).

As the “conclusive-sensory-evidence hypothesis” (CSE hypothesis), like the conceptions of Michotte and Kanizsa, is purely phenomenological in nature, it allows to “abandon the assumption that modal and amodal percepts are linked to the dichotomy between unoccluded and occluded scene regions” (Scherzer & Ekroll, 2015, p 11). Nevertheless, referring to this hypothesis often leads to very similar classifications, as the sensory evidence of certain characteristics is usually conclusive in regions of the proximal stimulus that correspond to geometrically optically visible parts of the scene and weak in regions of the stimulus corresponding to occluded parts.

The CSE hypothesis not only permits a consistent classification of completion phenomena, as will be shown later, but it is also appealing because it makes predictions about the possible meaning of modal and amodal percepts, namely, that the quality of a percept represents the conclusiveness of its sensory evidence. In this way, it may offer explanations for previously unexplained completion phenomena such as the ubiquitous occlusion illusion (Scherzer & Ekroll, 2015, p. 10). It is also compatible with most theoretical explanations for modal and amodal completion phenomena (e.g., Anderson, 2007; Anderson et al., 2002; Gerbino & Salmaso, 1985; Kellman et al., 2005; Kellman & Shipley, 1991; Kellman et al., 1998; Purghé & Coren, 1992; Ringach & Shapley, 1996; Singh, 2004; Tse, 1999b). In this article, however, we concentrate on developing consistent classification criteria for completion phenomena based on the CSE hypothesis. The hypothesis comprises two subhypotheses:

1. *The continuity hypothesis*: When modal and amodal percepts are decoupled from the dichotomy between unoccluded and occluded scene regions, the phenomenological attribute “modal/amodal” could be a continuous rather than a dichotomous variable that depends continuously on the conclusiveness of the sensory evidence (Scherzer & Ekroll, 2015, p 11). Modal and amodal impressions would then correspond to the two end points of the modal–amodal continuum. In other words: The presence of visual qualities may indicate conclusive sensory evidence of corresponding characteristics, their weaker presence weaker evidence, and their absence no evidence. Figure 11(a) shows gradations of the presence of brightness over space.
2. *Perceptual attributes are visual qualities*: Any visual attribute that can be perceptually present should be considered a visual quality of the same ontological status as brightness and color. Scherzer and Ekroll (2015) point out that, for example, the shape and contour of an object can be experienced as entirely compelling (i.e., perceptually present) in amodal displays, whereas brightness and color are not, and argue that it is therefore difficult to justify to consider the latter as visual qualities but not the former (ibid., p 11). This view broadens the concept of visual quality, which for Michotte was limited to local surface attributes such as brightness and color.

We propose to distinguish at least three classes of attributes of visual complements (and visual percepts in general):

- The “unity class,” including attributes such as unity, connectedness, and “common fate.”
- The “shape class,” including attributes such as contours and shape^16^ and thus, implicitly, depth.
- The “quality class,” including attributes such as specific features and characteristics.

At first glance, the distinction between these three classes is very similar to the “task-oriented taxonomy of visual completion” proposed by Yin (1998). However, its real potential only emerges in combination with the continuity hypothesis, as will be shown later. Furthermore, the three classes should be considered in a more abstract sense so that they can cover not only object completion but, for example, also motion completion and nonvisual completions.

Accordingly, attributes of objects include as follows:

- Object and surface unity
- Surface contour, object shape, and depth
- Surface qualities (e.g., brightness, color, texture, and specific material)

Attributes of motion—not attributes of a moving object!—include analogously:

- Motion connectedness
- Motion path and depth
- Motion speed and smoothness

From these two subhypotheses, some further implications can be derived:

1. *Sensory evidence is attribute-based*: The conclusiveness of the sensory evidence regarding one perceptual attribute in a specific region of the visual field may be different from that regarding a different perceptual attribute in the same region. It is therefore

possible that some of the attributes pertaining to an occluded region of the visual field may be experienced as phenomenally clearly specified and distinct (i.e., visible), whereas others may be not specified and distinct or poorly specified and distinct (i.e., invisible or vaguely visible, respectively). (Scherzer & Ekroll, 2015, p. 11)

Of course, the same may also apply to some attributes pertaining to unoccluded regions of the visual field.

With respect to the situation depicted in Figure 1, for example, there would be conclusive sensory evidence for the unity and exact shape and contour of the gray stimulus in the lower right, which is why it is clearly perceived as a (partly occluded) triangle. However, the sensory evidence for its specific surface color or texture under the black occluders would seem rather weak, and hence there is no visual impression of brightness or color (of the triangle) in this region.

1. *Sensory evidence may vary with the location*: The presence of certain perceptual attributes, that is, visual qualities, may vary abruptly or continuously across the visual field, depending on the conclusiveness of the sensory evidence of corresponding characteristics at different locations. For example, there are abrupt changes in the conclusiveness of the sensory evidence for the specific surface color of the triangle in the lower right of Figure 1: The evidence is conclusive in the unoccluded regions and very weak in the occluded regions. Continuous changes in the local sensory evidence would, for example, be expected regarding the surface color of the fuzzy figure shown in Figure 11(a).

The conclusiveness of the sensory evidence regarding global attributes such as the unity or the exact shape or contour also seems to depend on the local sensory evidences at critical locations in the visual field. If the local sensory evidence regarding one attribute is weak at some critical locations, its overall evidence cannot be very conclusive anymore. For example, the overall sensory evidence of a triangle in Figure 11(a) seems very weak because there is no local evidence for this solution in the upper region, although there seems to be conclusive local evidence of a horizontal contour and two oblique contour pieces at the bottom. In contrast, in accordance with the Gestalt principle of good continuation, there may be conclusive local evidence of some curved contour similar to that illustrated in Figure 11(b), which would result in a somewhat diffuse percept regarding the exact shape and contour of the figure.

#### Terminology

Scherzer and Ekroll (2015, p. 11) describe perceptual attributes based on conclusive sensory evidence as “phenomenally clearly specified and distinct (i.e., visible)” and perceptual attributes without or based on only weak sensory evidence as “not specified and distinct or poorly specified and distinct (i.e., invisible or vaguely visible, respectively).” Note the inherent continuous character of the phenomenological variable “strength of phenomenal specification.” Note also that any perceptual attribute can be characterized in the way described earlier, that is, the classification scheme is not limited to the added parts of completed objects.

#### The role of conclusiveness in the CSE hypothesis

The CSE hypothesis focuses on essential *phenomenological* aspects of the percept (i.e., the perceptual presence, or “visibility,” of different attributes) and the potential *functional* meaning of visual qualities (namely, as indicative of the reliability and robustness of the underlying perceptual inferences) in a broad sense. A key concept is the conclusiveness of the sensory evidence.

The important question of which stimulus characteristics provide conclusive sensory evidence is not part of the CSE hypothesis, but it builds directly on Rock’s (1983) sophisticated “logic of perception.” Rock’s conception is that “the final percept must conform to and be supported by the stimulus” (p. 132), that is, “the solution must account for the proximal stimulus” (p. 118), also for absences of parts or features that “should be present” (p. 123), it “must not entail contradiction” (p. 125), and “the proximal stimulus must contain what is implied by the solution” (p. 120; “stimulus support,” pp. 120–125). All this is essentially what the term “conclusiveness of the sensory evidence” is intended to cover, and it implicitly also includes the related common-cause, single/common-explanation, and rejection-of-coincidence principles mentioned by Rock (pp. 134–146).

The CSE hypothesis complements Rock’s conception with important aspects concerning visual qualities and their potential functional meaning. Applying the CSE hypothesis may therefore inspire informative deductions on the basis of pure phenomenology, as will be illustrated later. In particular, the continuity subhypothesis also offers an explanation for fuzzy or underspecified properties of the percept, for example, for the “incomplete” illusory triangle in Figure 11(a). This seems compatible with Rock’s (1983, p. 141) observation that “the more coincidence or otherwise unexplained regularity there is, the stronger the preference for an illusory-contour percept,” and that “the effect seems to be more immediate, ‘better’, stable, and irreversible” in specific cases than in others.

For example, one might wonder why the sensory evidence of a Kanizsa-like triangle as in Figure 15 (upper left) should be conclusive, even though this percept requires a figure-ground reversal and large sections of the contour are not contained in the stimulus. We follow Rock’s (1983, p. 140) view that “there must be a strong reason for this preference” and his line of argument that

the illusory-contour percept provides a single explanation for what would otherwise be unexplained coincidence: incomplete circles whose missing parts have edges aligned or collinear with those of other incomplete circles and the potential presence of a triangular figure in the white central region. (pp. 140, 141)^17^

According to the CSE hypothesis, the extraordinary salience of the triangle (its unity, shape, contour, and surface color) may be indicative of a great reliability of the underlying perceptual inferences.

#### Application: Classifying different phenomena

To illustrate the classification criteria, they will now be applied to some well-known phenomena.

*Kanizsa figures*: Many Kanizsa-like figures like the Kanizsa triangle (upper left of Figure 1) are clearly perceived as one unitary object. Their shape and contour are clearly visible, and their surface glows brighter than the same colored background. All attributes would therefore be regarded as phenomenally specified. This specification pattern is typical for complements called “modal” in the literature.

*Partly occluded objects and self-occlusion*: Partly occluded objects such as the triangle in the lower right of Figure 1 are clearly perceived as one unitary object and their shape and contour are visually clearly present, even in the occluded regions. All these attributes would therefore be regarded as phenomenally specified. However, no surface color is visually present in the occluded regions, which is why this attribute is regarded as phenomenally unspecified. This specification pattern and, according to the CSE hypothesis, the corresponding pattern of conclusiveness, is typical for complements called “amodal” in the literature. It also applies to cases of self-occlusion. Note, however, that any completion of partly (self-)occluded objects may also imply rather conclusive sensory evidence, for example, with regard to the surface color in certain regions of the proximal stimulus that correspond to geometrically optically occluded regions of the distal stimulus without being noticed, as with the static and dynamic occlusion illusion (Figures 13 and 14). See Scherzer and Ekroll (2015) for more details on that.

*Occlusion illusion*: In the static and dynamic occlusion illusion (Figures 13 and 14), the target object shows the typical characteristics of partly occluded objects as described earlier. Phenomenology and empirical findings indicate, however, that its surface color is also phenomenally specified, that is, visually present, in some occluded regions adjacent to unoccluded regions. Therefore, the CSE hypothesis would assume that there is conclusive sensory evidence regarding this attribute even in these regions. Scherzer and Ekroll (2015, p. 10) discuss potential sources of sensory evidence in these stimuli.

*Virtual figures*: Collections of dots as shown in Figure 10 seem to form one unitary object with a visually distinct shape. However, unlike with partly occluded objects, there is no impression of a contour, a surface or any other “indefinable material” at all. In this respect, virtual figures differ significantly from partly occluded objects.

*Integration*: Michotte et al. (1964/1991, pp. 145, 146) describe isolated stimulus elements that are perceived as one broken figure (like the triangle in Figure 2(b)) as an example of integration, “as a result of which it has the immediate appearance of being formed of two parts separated from each other.” Resembling virtual figures, the specification pattern here seems to be similar to cases of “classical amodal completion” of partly occluded objects. They note, however, that “integration [originally: intégration] is not sufficient on its own in such a case to produce the completion [originally: complément]; other conditions have to be satisfied,” namely, that the “line of demarcation” must be perceived as belonging to the contour of a screen. Unlike classical amodal complements, however, the impression of the unity of separated stimulus elements—an apparent paradox—varies continuously with critical stimulus characteristics (e.g., the relative length of the perimeter actually displayed; Bobbit, 1942) and even though the overall shape of the broken whole is clearly specified (as in the case of classical amodal complements), the contour is *visibly* interrupted at certain locations. This specification pattern is clearly different from that of classical amodal complements. According to the CSE hypothesis, the perceived degree of object unity, the clearly specified overall shape, and the missing contour at certain locations reflect the conclusiveness of the sensory evidence regarding the corresponding attributes. This pattern of conclusiveness could be explained by the fact that the gaps cannot be attributed to an external factor (e.g., the presence of an occluding screen), which excludes the interpretation of an unbroken contour. This would be consistent with Michotte et al. (1964/1991, pp. 145, 146) and Rock (1983).

**Figure 16.**
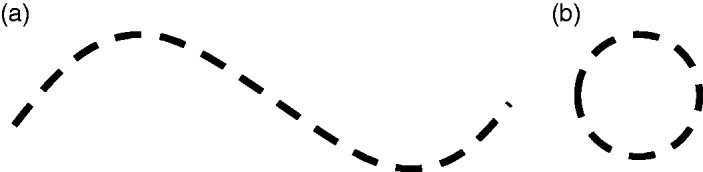
Figures with broken contours.

*Broken versus virtual figures*: One may wonder whether percepts like those evoked by Figures 2(b) and 16, which are based on integration in Michotte et al.’s (1964/1991) sense, could also be regarded as virtual figures like collections of dots as shown in Figure 10. This may be suggested by their obvious similarity: Both broken contours and dot collections can form one unitary object with a visually distinct shape, although they are only sparsely defined in the stimulus. They also share the property that they have no surface qualities. However, broken figures are also phenomenally noticeably different from virtual figures: While the latter lack a perceived contour, it is clearly visible in broken figures, albeit it has salient gaps. Thus, broken figures are, in a sense, more fully specified than dot figures, but at the same time, they appear as less “complete,” which may seem paradoxical at first sight. According to the CSE hypothesis, however, this phenomenal incompleteness of broken figures could be explained by the “unexplained” abrupt absence of the (otherwise present) contour at certain locations in the stimulus, which considerably weakens the sensory evidence of completeness. No such detrimental effect can occur with virtual figures, as they have no contour at all. This illustrates that additional phenomenal attributes as such do not necessarily increase the overall completeness, as they also open up the possibility of an incompleteness in specification with a corresponding phenomenal effect.

*Grouping*: The perceptual integration of isolated stimulus elements into wholes and perceptual completions can also be understood as different kinds of grouping. Wagemans (2018, pp. 833–838) recently suggested to distinguish between five types of grouping: clustering (of sets of elements that share one or more properties), segregating (between two of those subsets), linking (of elements in a specific way, e.g., pairwise coupling to lines or contours), layering (segregating with figure/ground attribution), and configuring (organizing individual elements in larger, structured wholes or Gestalts with configural properties, e.g., in closed shapes with high degrees of regularity or in familiar objects). All grouping types have in common that (by definition) the unity of the grouped elements must phenomenally specified to some degree. All grouping types except clustering also require the phenomenal presence of some kind of border, for example, the shape or (parts of) a contour. Taking the continuity subhypothesis into account, it may be interesting to consider that the transitions between different types of grouping may be continuous too (e.g., in case of “diffuse” segregation). Layering and configuring additionally seem to require the phenomenal presence of a shape. In general, grouping does not necessarily depend on the phenomenal presence of (contour/surface) qualities. On the other hand, quality properties as such can serve as a grouping factor. These considerations suggest that the proposed classification scheme might help to refine the existing types of grouping from a phenomenological view.

*Tunnel effect*: The connectedness of motion in the tunnel effect (Figure 8), the motion path, and specific qualities like motion speed and smoothness depend on the exact experimental parameters (Burke, 1952), but it is possible to find parameters such that all motion attributes appear perceptually clearly specified. The specification of the attributes of the temporarily occluded carrier object is, however, similar to that of partly occluded objects.

Finally, we would like to apply the classification criteria to a few phenomena that have recently been published in this special issue of *i-Perception* on amodal completion. Tse (2017a) presents dynamic stimuli that evoke “classical” modally and amodally completed volumes, which undergo a deformation. Tse (2017b, p. 1) also presents Kanizsa-like stimulus pairs which, binocularly fused, “can give rise to a percept of 3D curved, nonclosed illusory contours and surfaces.” They are phenomenally very similar to “ordinary” Kanizsa-like figures but are in addition smoothly interpolated in depth. Not surprisingly, most of the other articles in the collection deal with new findings on (static or dynamic) “classical amodal completion,” that is, with partly occluded objects as already discussed earlier (Anstis, 2018; Chen, Schnabl, Müller, & Conci, 2018; Ekroll, de Bruyckere, Vanwezemael, & Wagemans, 2018; Kiritani, Kawasaki, & Chang, 2018). We have therefore chosen three different examples, which demonstrate particularly well the potential of the aforementioned classification scheme with regard to interpret specific aspects of various phenomena:

*Thin building illusion*: Ekroll, Mertens, and Wagemans (2018, p. 1) show that “a tall pillar with a triangular base evokes radically different three-dimensional (3D) percepts depending on the vantage point from which it is observed,” namely, from a flat surface to a pillar with a rectangular base to a pillar with a triangular base. Their findings are related to previous works on volume completion (Tse, 1999b), self-occlusion (van Lier & Wagemans, 1999), and fuzzy regularities (van Lier, 1999). Interestingly, there is a large variation in the data at certain viewing positions, where the conclusiveness of the sensory evidence for the shape on the back seems rather weak.

*Shape without contour*: The Phillips painting in Koenderink, van Doorn, and Wagemans (2018, Figure 2, left painting) is a great example of how subtle the differences between similar stimuli can be and how well they are captured by the proposed classification scheme. The painting shows a Kanizsa-like figure, namely, a woman whose contour is not physically present in the stimulus but is indicated by very few line endings of a spider web partly occluded by the woman’s body. Koenderink et al. (2018, p. 2) call these contours amodal, that is, “a perceived contour (a *quale*) for which there does not exist a relatively sharp, relatively straight transition between two more or less uniform, extended, and contrasting regions in the stimulus (*a property of the image*).” In this way, they link the phenomenal (subjective) impression with the (objective) stimulus properties. In contrast, the proposed classification scheme intendedly refers only to phenomenal properties. The woman in the painting would therefore rather be characterized as follows: Her unity and shape are visually quite clearly specified, which is also supported by the experimental results of Koenderink et al. (2018, Figure 4, left panel). Unlike a Kanizsa triangle, however, her contour seems only slightly indicated (if at all).^18^ Her dress is visibly colored, that is, it contains surface qualities, but the brightening effect is much weaker than with a Kanizsa triangle.

*Ensemble perception*: Haberman and Ulrich (2019, p. 2) present new experiments on ensemble perception, which is the phenomenon that “in the face of overwhelming information, the visual system can exploit the redundancy of natural scenes by representing their summary statistics.” They compared sets of incomplete faces that either were amodally completed behind horizontal bars or were presented in a fragmented way in the foreground and found that “the ensemble representation of amodally completing sets was significantly better than the fragmented sets” (p. 1). According to the proposed classification criteria, the task-relevant perceptual attributes such as unity, shape, and contour (and perhaps to a certain degree surface qualities as well) were visually much more specified in the completion condition than in the fragmented condition. It would therefore be possible that the goodness of ensemble representation actually depends on the conclusiveness of the sensory evidence regarding these attributes in the individual set elements. This is consistent with Haberman and Ulrich’s (2019, p. 8) finding in Experiment 2 that “[a]modal completion may not actually allow the visual system to recreate the missing information, but rather support a best-guess heuristic, akin to visual completion.”

## Conclusion

The common dichotomous distinction between modal and amodal percepts is problematic in phenomenological, empirical, logical, and theoretical terms. Scherzer and Ekroll’s (2015) proposal to regard the phenomenological variable “modal versus amodal” as continuous, and perceptual attributes such as unity and shape as visual qualities on a par with brightness and color, allows a considerably more precise description and classification of visual (completion) phenomena. The CSE hypothesis related to these assumptions states that the phenomenal presence represents the conclusiveness of the sensory evidence. It is not restricted to visual completion phenomena but can be applied to visual percepts in general. In principle, it could even be generalized for application to nonvisual perception.

## Notes

1 This concept was also applied to nonvisual domains (see, e.g., Bregman, 1981, for auditory perception). In this article, however, we focus only on visual perception.

2 Authors’ note: Illusory contours, surfaces, figures, and so on are sometimes also called “subjective,” “anomalous,” or “cognitive” (Kanizsa, 1979, p. 192). In this article, we use the most common term “illusory.”

3 The origin of the nowadays common, but in our view inaccurate translation, is not clearly reconstructable for us. According to a research with Google Scholar, the English translation “amodal completion” seems to appear for the first time in the “References” section of an unpublished manuscript by Gibson (1966). Its first occurrence in a published article text appears to be in Kaplan (1969), who interestingly enough uses both the terms “amodal completion” and “amodal perception” (p. 197). We suspect, however, that Kanizsa (1972, 1975, etc.) has established the term “amodal completion” in its current use through his numerous publications on this subject in English. This is in line with van Lier and Gerbino (2015, p. 294, original italics), who find that, “[b]y describing amodal completion as a process instantiated by stimulus-defined incompleteness […], Kanizsa (1954, 1955/1987) went beyond the phenomenological notions of *unsichtbar vorhanden* (invisibly present, Metzger, 1936, Chapter 8) and *donnée amodal* (amodal datum, Michotte and Burke, 1951).” Authors’ note: “Donnée amodal” can also be translated as “the amodally given.”

4 Authors’ note: Instead of the photometric measure of luminance, it would probably be more accurate to speak of visual qualities such as brightness here.

5 We have not tested it experimentally, but it seems also plausible to us that stimuli as in Figure 2(b) may lead to the (perhaps weak) percept of an amodally completed triangle behind a modal illusory vertical white rectangle or strip, provided the position of the gaps is consistent with this interpretation.

6 Note that what we call “perceptual transparency” is occasionally also referred to as “apparent transparency” in the literature, maybe sometimes “to stress that real transparency […] is neither necessary nor sufficient to support a transparency percept” (Gerbino, 2015, footnote 4, p. 415). Although both apparent transparency and perceptual transparency are often phenomenally similar, we make a clear distinction between them to highlight the differences in the stimulus conditions.

7 The usual view today that the underlying process is perceptual and not imaginative neither supports nor contradicts Nanay’s (2018) claim that amodal completion is a form of mental imagery, provided that mental imagery, in distinction to imagination (ibid., p. 9), is conceived as a form of perceptual processing as in Nanay’s sense.

8 We would like to leave open here whether the illusory occluder causes the amodal complement (analogous to the “causal hypothesis” mentioned in van Lier & Gerbino, 2015, p. 296, according to which amodal completion causes modal completion) or whether the percept is the “simultaneous result” of an underlying problem-solving process.

9 Wagemans and d’Ydewalle (1989, p. 290) later demonstrated that “amodal completion [of the carrier object in the tunnel effect] can be enhanced or inhibited by information specified over time,” that is, by prior supportive or contradictory stimulus information. Their experimental results suggest that even the memorized stimulus history can influence amodal completion.

10 Although not mentioned by Michotte et al., the perception of “something” in empty space sounds similar to reports of subjects in earlier investigations of von Karpinska (1910) who used binocularly fused images of outline figures. Their percept seemed to consist of “something” whose material was rather “airy, transparent,” like “gelatin, glass, a very thin, indefinable substance, [or] compressed and crystallized air” (Schumann, 1920, p. 228, our translation). See Mausfeld (2010) for further reading (but also note Möller, 1925).

11 One could argue that a transparent indefinable substance, clearly different from, for example, a Kanizsa triangle, has no material quality and therefore no modal quality. However, just because there is no appropriate verbal description for this (“undefinable”) does not mean that there is no visually clearly defined quality. Otherwise, it would be unclear how the indefinable substance could be phenomenally distinguished from its surround (see the previous footnote).

12 Unlike brightness and color, however, they are not limited to the visual domain.

13 Michotte et al. (1964/1991) would probably (only) speak of integration instead of completion/complement in these cases (see their discussion on the broken triangle in their Figure 3.3, pp. 145, 146, and our discussion of Figure 2(b)). For more details, see our thoughts on grouping (p. 27).

14 Note that Michotte et al. (1964/1991, p. 164) already mentioned, however, in the context of the tunnel effect and with reference to the motion percept rather than the object percept, that “these formal properties of the [amodal] completion [originally: ces propriétés formelles du complément] can bring about apparent changes in the uncovered parts of the two trajectories that it links.”

15 It should be noted that in the occlusion illusion, the sensory qualities of a certain proportion of the added parts are present (“partial modal complement”; Palmer et al., 2007). It is yet unknown whether amodal complements are *always* accompanied by a partial modal complement or whether this only occurs under specific stimulus conditions.

16 Note that the perceived shape and contour, though closely related, are not interchangeable. Consider, for example, Figure 2(b), which evokes the clear percept of the shape of a triangle, although its contour is visibly broken into two isolated angles. Conversely, a contour alone is generally not sufficient to form a shape.

17 Rock (1983, p. 121) believes that, in the case of Kanizsa-like illusory figures, “the perceptual system constructs or invents a subtle difference in color to support the overall solution.”

18 This deviates from the characterization given by Koenderink et al. (2018) who regard the contour as a *quale*. We believe that what is visually present, that is, a *quale*, is actually the shape, not the contour.
